# Spatiotemporal Heterogeneity of Lung-Deposited Surface Area in Zurich Switzerland: Lung-Deposited Surface Area as a New Routine Metric for Ambient Particle Monitoring

**DOI:** 10.3389/ijph.2023.1605879

**Published:** 2023-06-29

**Authors:** Jacinta Edebeli, Curdin Spirig, Stefan Fluck, Martin Fierz, Julien Anet

**Affiliations:** ^1^ Center for Aviation, School of Engineering, Zurich University of Applied Sciences, Winterthur, Switzerland; ^2^ Naneos Particle Solution GmbH, Windisch, Switzerland

**Keywords:** particle surface area, UFP exposure, lung-deposited surface area (LDSA), ambient UFP monitoring, ultrafine particles (UFP)

## Abstract

**Objective:** To assess the spatiotemporal heterogeneity of lung-deposited particle surface area concentration (LDSA), while testing the long-term performance of a prototype of low-cost-low-maintenance LDSA sensors. One factor hampering epidemiological studies on fine to ultrafine particles (F-to-UFP) exposure is exposure error due to their high spatiotemporal heterogeneity, not reflected in particle mass. Though LDSA shows consistent associations between F-to-UFP exposure and health effects, LDSA data are limited.

**Methods:** We measured LDSA in a network of ten sensors, including urban, suburban, and rural environments in Zurich, Switzerland. With traffic counts, traffic co-pollutant concentrations, and meteorological parameters, we assessed the drivers of the LDSA observations.

**Results:** LDSA reflected the high spatiotemporal heterogeneity of F-to-UFP. With micrometeorological influences, local sources like road traffic, restaurants, air traffic, and residential combustion drove LDSA. The temporal pattern of LDSA reflected that of the local sources.

**Conclusion:** LDSA may be a viable metric for inexpensively characterizing F-to-UFP exposure. The tested devices generated sound data and may significantly contribute to filling the LDSA exposure data gap, providing grounds for more statistically significant epidemiological studies and regulation of F-to-UFP.

## Introduction

Particulate matter (PM) is an air quality concern, hence the measures for limiting exposure to PM. Present regulations are mass-based, focusing on PM size classes below 10 µm [1, 2]. The commonly monitored PM size classes are PM1, PM2.5, and PM10, including PM of diameters below 10 µm in PM10, to below 1 µm in PM1. In Switzerland, the PM2.5 annual average concentration limit is 10 μg/m^3^ and for PM10, 20 μg/m^3^ [2]. The new WHO guidelines recommend annual average limits of 5 μg/m^3^ for PM2.5 and 15 μg/m^3^ for PM10 [3].

Regulations and technological advancements have promoted or caused decrease in PM mass and PM number concentrations (PM_mass and PNC, respectively) [4, 5]. The large decrease in PM_mass is partly driven by a shift in PM size towards the PM1 class, while decrease in PNC has been slower [4, 6, 7]. Within the PM1 class, quasi-ultrafine particles (qUFP, diameter < 500 nm) to ultrafine particles (UFPs, diameter < 100 nm) are deemed most relevant for PM health impacts [8–15], with traffic being the main source of urban UFPs [16, 17]. Research shows that UFPs and qUFPs contribute up to 90% of total ambient PNC, while UFPs make less than 1 μg/m^3^ of ambient PM_mass [6, 9, 18–20]. Therefore, the true health implication of PM exposure may not be adequately assessed and mitigated with mass-based strategies. There are still no regulations for—or standardized monitoring of—UFPs due to limited data and poor understanding of their distribution, composition, and impact [21, 22]. This limitation may be minimized through continued research. The research community is also considering expanding the UFP range to include particles up to 500 nm [21]; from here on, we will refer to qUFPs to UFPs simply as UFPs.

UFPs have high lung deposition, retention and penetration efficiency, and high specific surface area (SSA) [8–11, 15, 18, 23]. This high SSA allows for interactions with lung surfaces, adsorption and transport of co-pollutants, and surface reactions [11, 12, 15]. Studies have shown strong relationships between health impacts and the surface area (SA) of particles deposited in the lungs, concluding that SA is one of the most relevant metrics for the health impact of UFPs [8, 9, 11, 12, 15, 24, 25]. However, present research on UFP is dominated by PNC measurements [18], and there is no routine monitoring of particle SA in most countries.

A metric for particle SA exists, lung-deposited surface area concentration, LDSA [µm^2^/cm^3^], which is the SA of particles deposited in the lungs [26–29]. Measuring the active SA of particles, LDSA provides a proxy for particle reactivity and potential for interaction with the lungs [9]. With particle number size distribution (PNSD) and charge as measured, e.g., with an Electrical Low Pressure Impactor (ELPI), one can also calculate LDSA [30, 31]. However, the observed high heterogeneity in the time and space of UFPs [19, 32–35] demands monitoring through a network of sensors [10, 35, 36]; yet a network of particle counters for PNC or ELPIs would be expensive [37]. As not all particle sizes are deposited in the lungs [9, 23, 38], LDSA measurements that target the dominant fraction of ambient particles with high deposition and penetration efficiency in the lungs, such as with low-cost diffusion chargers, will be more appropriate [26–29].

There are two classes of LDSA: alveolar (A-) and tracheobronchial (Tb-) LDSA [28, 39]. A-LDSA, the more commonly measured LDSA, refers to the LDSA in the alveoli in the lungs, with direct access to the bloodstream; Tb-LDSA refers to the LDSA in the Tb-region of the lungs [9, 28, 39, 40]. Current A-LDSA measurement methods target particles in the 10 to ∼400 nm size range [9, 10, 15, 28, 39], and existing measurement devices are accurate between 20 and 400 nm [10, 37, 41]. This size range coincides with the highest deposition range in the A-region of the lungs [12]. In this study, we focus on A-LDSA, referred to as LDSA from here on.

Research shows that LDSA captures the high spatiotemporal heterogeneity of ambient UFP [10, 39, 40, 42]. The variability in LDSA is strongly dependent on local emission sources, local and regional meteorology (especially wind), and distance from the source [10, 39, 40, 43]. LDSA is primarily attributed to road traffic and residential combustion, reflecting the temporal patterns of these sources [10, 30, 39, 43]. Therefore, observations show LDSA peaks during traffic rush-hours and periods of residential activities (evenings and weekends), especially under a low and stable atmospheric boundary layer as in the winter and night-time [10, 30, 39, 43]. Some studies have also observed photochemical new particle formation (NPF) and/or secondary aerosol formation (SAF; nucleation and particle growth) contributing to daytime increase in LDSA, especially with high insolation and low pollution [10, 30, 39, 40, 43, 44]. In general, the highest LDSA has been observed in the winter, with residential heating and lower atmospheric dilution [39, 43]. Kuula et al., however, observed elevated LDSA in the warmer months in Finland, attributed to possible SAF [10]. Research has observed correlations between LDSA and nitrogen oxides (NOx), black carbon (BC) and PM mass metrics, dependent on the sources of PM and seasons, with higher correlations in the colder seasons [10, 22, 39, 42, 43].

With limited data on LDSA and the absence of a long-term network for LDSA, we set out to measure LDSA in a network of 10 prototype low-cost-low-maintenance A-LDSA sensors. Our objective was to investigate the variability of LDSA in Zurich, Switzerland and identify the driving factors, while assessing the performance of these prototype sensors. Here, we present our observations of LDSA in this network. We also present an assessment of LDSA amongst other PM metrics.

## Methods

### The Network of Devices

Ten LDSA devices were deployed at urban (5, including the city center and by major roads), suburban (2), and rural (3) locations in Zurich, Switzerland. Supplementary Table S1 presents a description of each station’s surroundings. The Brütten station provides the regional background signal, being the most remote station, with negligible local anthropogenic emissions. Two campaigns were conducted at all 10 locations between 2020 and 2022. The first campaign was conducted between 7 May and 15 August 2020, and the second campaign commenced in April 2021 at Dübendorf (suburban), Rosengartenstrasse (urban/major road), Schimmelstrasse (urban) and Stampfenbachstrasse (urban), while measurements at other stations commenced in February 2021 until February 2022. For further assessments below, we focused on the 2021 to 2022 campaign to assess long-term continuous trends.

### Instrumentation

We measured LDSA using the prototype of an A-LDSA-calibrated sensor built by naneos particle solutions GmbH Switzerland (naneos), the developers of the established Partector 2, an LDSA measuring device, which can be calibrated for A- or Tb-LDSA [28]. The measurement principle and cell of the deployed devices are the same as those of the Partector 2, where the active SA of particles are detected by diffusion charging [28, 45]. Like the Partector 2 for A-LDSA, the devices are accurate between 20 and 350 nm. The concentration accuracy range is from the detection limit (LOD; 2 μm^2^/cm^3^) to 20,000 μm^2^/cm^3^ [28]. In addition to LDSA, temperature and relative humidity (RH) within the device, air flow, fan current, and electrical gain are reported by the instrument. The devices are temperature regulated to keep the RH around 40%. The electrical gain, fan current, and air flow are relevant for assessing the performance of the device and the data quality. All data logged by the devices were remotely accessible. The devices were calibrated with a reference scanning mobility particle sizer (SMPS) by naneos using similar procedures for the Partector 2 [28, 29, 45], and showed good qualitative agreement with PNC in the field ([45]; Supplementary Figure S1). In addition, co-location checks before and after the 2021–2022 campaign confirmed the precision of the devices. Relative standard deviation was 2.7% (*n* = 10) before deployment [Supplementary Figure S2 (right)]. The precision remained good after a year without maintenance on the functioning devices (relative standard deviation = 4.3%, *n* = 9), excluding a weather-damaged device [Supplementary Figure S2 (left)].

### Road Traffic and Traffic Co-Pollutants, and Meteorology Data

Traffic and meteorology influence LDSA [10, 39, 40, 43]. To assess measured LDSA, hourly traffic counts, traffic co-pollutant concentrations, and meteorological data were obtained from different monitoring agencies. The total traffic counts in both directions were obtained for the main road next to the Rosengartenstrasse station (four lanes with a 50 km/h speed limit) and the Opfikon Balsberg (Balsberg) station (six lanes with varying speed limit between 80 and 100 km/h) from Zurich’s Civil Engineering office [46] and the Cantonal Federal Roads office in Switzerland for Zurich [47]. Traffic co-pollutant concentrations (NOx and PM_mass for PM2.5 and PM10) were obtained from local monitoring stations from the OSTLUFT online database [48]. In addition, PNC were obtained from the National Air Pollution Monitoring Network (NABEL), for 2021 at Kaserne [49] and from Zurich’s Department of Environment and Health at Stampfenbachstrasse [50].

Hourly weather data were obtained from MeteoSwiss [51], where available. Rümlang and Brütten do not have local weather stations. As a result, weather data from Reckenholz were applied to Rümlang, and those for Kloten Feld (Kloten) to Brütten. We are aware of the shortcoming of the missing meteorological measurements: Reckenholz’s weather data may be applicable to Rümlang, which is ∼1.4 km from the weather station, but the Kloten data are less applicable to Brütten due to the distance (∼5.1 km), the topography (elevation difference of ∼150 m) and the surroundings (lower surface roughness in Brütten).

### Data Preparation

We obtained LDSA data at a frequency of one every 10 s in the 2021–2022 campaign. Due to occasional missing data and spikes in electrical gain in some devices, some steps were taken to clean up the datasets.

#### Electrical Gain Correction

The electrical gain of the measurement cell of all devices was measured by naneos under controlled conditions (*Gain*_*lab*_). Deviations from this *Gain*_*lab*_ may cause deviations in LDSA not related to the actual LDSA. As a result, a correction using Eq. 1 was applied to all datasets. Some devices developed some irregularities in the measured gain (*Gain*_*measured*_) leading to single spikes. These spikes in the *Gain*_*measured*_ were smoothed out, using the average between the gain before and after the spike, before conducting the gain correction.

$${LDSA}_{gain\_corrected}=\frac{{LDSA}_{measured}\times {Gain}_{lab}}{{Gain}_{measured}}$$ (1)

#### Flow Correction

An electrical fan drove the flow rate through the tested devices. The fan’s performance was monitored as the fan’s current, and a flow sensor measured the flow. Unfortunately, the flow sensor became clogged during the long campaign in some devices. The true flow through the instrument was calculated post-campaign using a flow correction method from naneos, though the flow does not strongly affect the device’s signal [45].

The final clean-up involved removing unreliable data. Data were dropped where the difference between the measured data and the corrected data exceeded 30%. Apart from Reckenholz (4%), Schimmelstrasse (0.3%) and Kloten Feld (<0.1%), data loss was below 0.006% for the 2021–2022 campaign (Supplementary Table S3). In addition, due to weather damage to the Reckenholz device, unreliable data in July and August and from November onward were removed from further analysis; this was the only device damaged during the campaign. Where data were below the LOD of 2 μm^2^/cm^3^ [28], they were assigned the LOD. The percentage of data below the LOD ranged from 0.8% at Rümlang (rural) to 0.1% at Rosengartenstrasse (urban/roadside). Those greater than the upper limit of 20,000 μm^2^/cm^3^ [28], which were two values at Stampfenbachstrasse, were assigned the upper limit. For further data analyses, we used hourly averages of LDSA for agreement with the time resolution of other datasets. Adjustments to LOD and the maximum limit changed the means for the overall sampling period by −0.1–1.8 μm^2^/cm^3^ and the medians by −0.2–1.5 μm^2^/cm^3^ across all stations.

#### Data Analyses

Data processing and analyses were conducted using Python version 3.7. For statistical analyses, we primarily used Scipy, a python package for scientific computing.

a. We used boxplots to visually represent the statistical distribution of LDSA at the different stations for the duration of the deployment.
b. To visually represent the spatial distribution with wind speed and direction, we used pollution roses. The pollution roses present the mean of LDSA within a grid of wind speed (10 bins) and direction (30 polar bins).
c. We estimated background LDSA as the rolling 10th percentile over a period of 6 hours (background LDSA). Here, we assessed the statistical difference between LDSA at the remote background location (Brütten) and the estimated background at other locations using independent sample t-tests, not assuming equal variance.
d. Correlations were assessed as Spearman’s rank correlations and/or Pearson’s correlation.

## Results

### Statistical Distribution of LDSA

**FIGURE 1:**
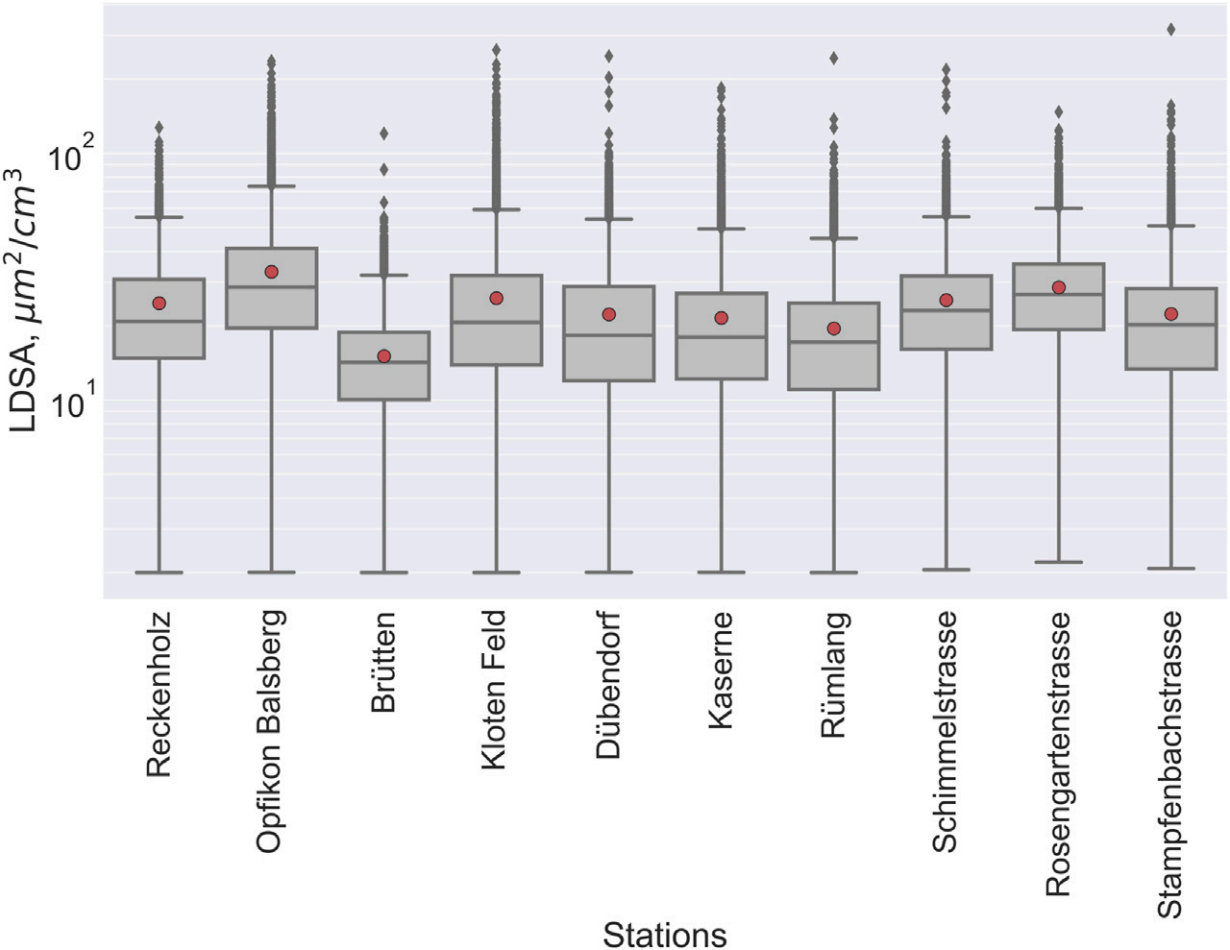
Boxplots showing the distribution of measured between lung-deposited surface area concentration (LDSA) during the 2021 to 2022 campaign. The vertical axis is in log scale. The boxes span the first and third quartiles, with the median line indicated; the whiskers span ±1.5 interquartile range, outliers (grey diamonds) fall outside the whiskers; red circles are the mean (Zurich, Switzerland. 2022).

Boxplots in Figure 1 present the distribution of LDSA hourly averages (log scale). LDSA ranged from the LOD (2 μm^2^/cm^3^) at all stations to 317 μm^2^/cm^3^ at Stampfenbachstrasse (Supplementary Table S4). The lowest median was at Brütten (14 μm^2^/cm^3^), while the highest median was at Balsberg (29 μm^2^/cm^3^). The mean at each station was within 1 and 5 μm^2^/cm^3^ higher than the respective median. High absolute difference between the mean and the median indicates the presence of outliers.

### Spatiotemporal Variability in Observed LDSA

#### Spatial Variability and Meteorology (Wind)

**FIGURE 2:**
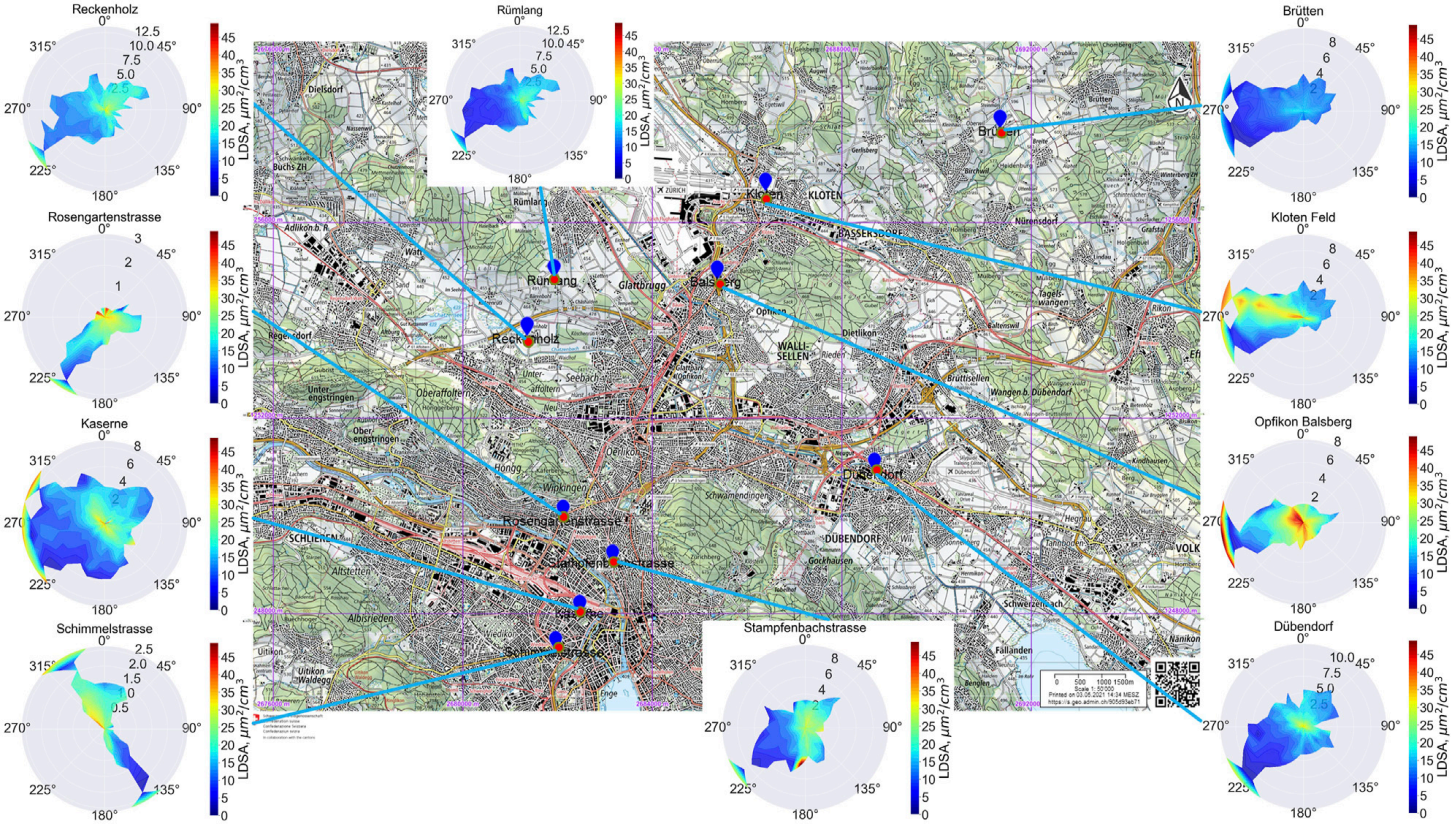
Pollution roses at the sampling locations showing the distribution of hourly lung-deposited surface area concentration (LDSA) with wind direction and speed (m/s) for the second campaign from 2021 to 2022. The colourmap scale goes from 0 (dark blue) to 50 (dark red) μm^2^/cm^3^. 30 wind speed bins were used. The base map was obtained from the Swiss Federal Geo portal [geo.admin.ch, scale: 1:50,000 (m)] (Zurich, Switzerland. 2022).

Figure 2 presents LDSA pollution roses at the different stations, showing LDSA (colourmap) as a function of wind speed and direction. High concentrations occurred at low wind speeds at stations close to intense local sources of pollution. Wind speed between 4 and 7 m/s was generally associated with lower concentrations; higher speed blowing over high pollution sources was associated with high LDSA. This phenomenon is especially visible at Balsberg; high wind speeds from the west transported pollutants to the station.

#### Temporal Variability

With measurements starting in February 2021, LDSA was measured at most stations through all four seasons. 2021 had an unusually cool and wet summer and a relatively dry fall [Supplementary Figures S3, S4 (bottom right)]. Seasonal LDSA distributions in Supplementary Figure S4 reflect this seasonal weather pattern, showing highest median in the fall at most stations, dominated by high night-time concentrations (Supplementary Figure S5). There were however often more outliers in the winter at most stations (Supplementary Figure S4).

##### Seasonal Diurnal Patterns

Here, we present the seasonal diurnal patterns in case studies:

###### Remote and Background LDSA

Brütten is the most remote station in this study. Here, there was small increase in LDSA in the morning on weekdays (which decreased in the late afternoon to evening in the spring and summer), with a small increase again at night [Supplementary Figure S6 (bottom left)]. In the fall and winter, LDSA increased again in the late afternoon to night-time (20:00 to 06:00 next day) and was higher than in the morning to early afternoon. Weekend daytime (06:00 to 20:00) LDSA was relatively flat and highest in the summer.

Estimated background LDSA at other stations was highest at night-time compared to the early hours of the morning. The background LDSA readings across all stations were highly correlated with each other (Supplementary Figure S7). T-tests showed that the means of the background LDSA readings were significantly different from the mean of the total LDSA at Brütten (Supplementary Table S5), though with high Spearman’s rank correlations (*ρ*), ranging from 0.73 to 0.78 (Supplementary Figure S7).

###### LDSA and Traffic Co-Pollutants

**FIGURE 3:**
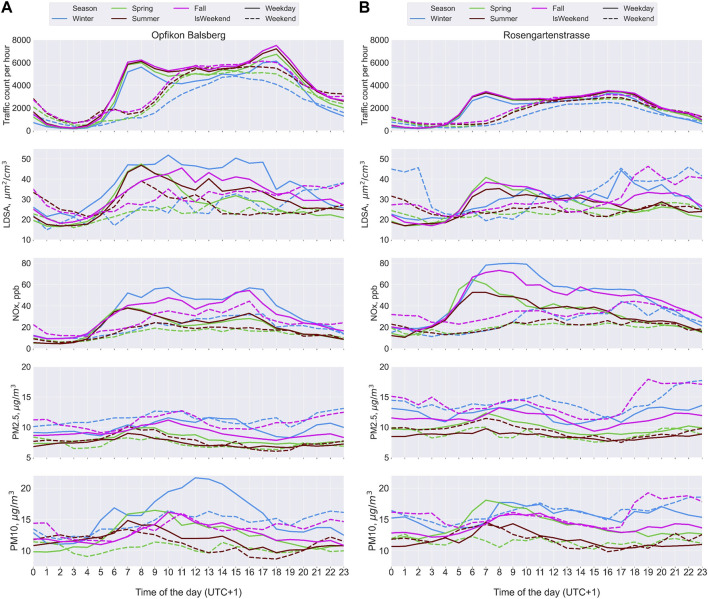
Seasonal diurnal pattern in median traffic counts, between lung-deposited surface area concentration (LDSA), Oxides of Nitrogen (NOx), Particulate matter of aerodynamic diameter less than 2.5 microns (PM2.5), particulate matter of aerodynamic diameter less than 10 microns (PM10) at Opfikon Balsberg [Balsberg, **(A)**] and Rosengartenstrasse **(B)** in 2021 (Zurich, Switzerland. 2022).

By major roads (Rosengartenstrasse and Balsberg), LDSA, like NOx, tracked traffic diurnal patterns, especially at Balsberg (Figure 3). The lowest traffic was observed in the winter, though with highest pollution at both stations. Despite Balsberg’s higher traffic volume than Rosengartenstrasse, apart from daytime LDSA and winter PM10, median emissions were higher at Rosengartenstrasse than at Balsberg. In the summer and spring afternoons, the median concentrations did not reflect the second traffic peak, except NOx at Balsberg. Especially at Rosengartenstrasse, winter and fall LDSA, PM2.5 and PM10 (on weekends) increased again at night-time. In each season, weekend concentrations stayed lower than weekdays during the day, except PM2.5.

At Rosengartenstrasse and Balsberg, NOx, NO and NO_2_ had the highest Pearson’s correlations with traffic and good correlation with LDSA, especially in the winter (Supplementary Figures S8, S9). NOx correlations with traffic ranged from 0.46 to 0.53 at Balsberg and 0.3 to 0.53 at Rosengartenstrasse. LDSA had lower correlation with traffic than NOx, 0.33 to 0.35 at Balsberg and 0.28 to 0.35 at Rosengartenstrasse. NOx correlations with LDSA ranged from 0.56 (summer) to 0.81 (winter) at Balsberg, and 0.44 (summer) to 0.77 (winter) at Rosengartenstrasse. PM10 and PM2.5 had little to no correlation with traffic and some correlation with LDSA. PM2.5 correlations with LDSA were 0.37 (fall) to 0.54 (winter) at Balsberg, and 0.34 (winter) to 0.5 (spring) at Rosengartenstrasse. PM10 correlations with LDSA were 0.33 (spring) to 0.52 (winter) at Balsberg, and 0.37 (winter) to 0.55 (spring) at Rosengartenstrasse.

###### Potential Airport Influence at Kloten Feld (Kloten)

**FIGURE 4:**
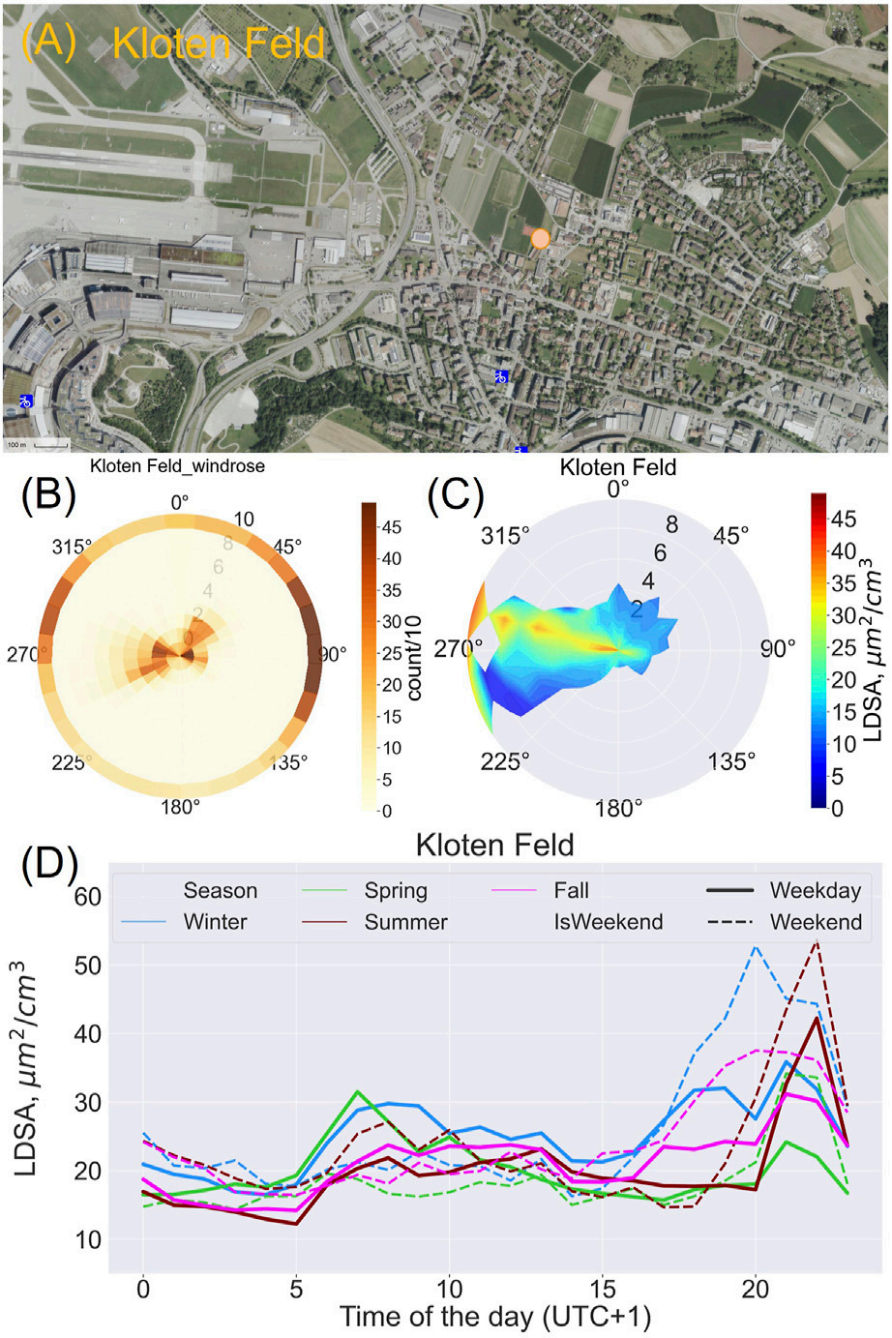
Lung deposited surface area concentration (LDSA) at Kloten: **(A)** The location of the Kloten Feld station on the map [https://www.maps.stadt-zuerich.ch/ creative-commons-Zero license (CC0), scale: 1:4,000 (m)] (Zurich, Switzerland. 2022); **(B)** Wind rose at Kloten; Radii are wind speeds, colors are the number of occurrences of wind from a direction (count) divided by 10; degrees are wind direction (Zurich, Switzerland. 2022); **(C)** Pollution rose of between lung-deposited surface area concentration (LDSA) in the 2021 to 2022 campaign; 30 levels in angles and 10 levels in wind speed, showing the influence of local polluting sources at low wind speed, and the transport of pollution at high wind speeds; radii are wind speeds, colors are average LDSA concentration; degrees are wind directions (Zurich, Switzerland. 2022); **(D)** Seasonal diurnal pattern of median lung-deposited surface area concentration (LDSA) at Kloten (Zurich, Switzerland. 2022).

The Kloten station is less than a kilometer southeast of the Zurich airport and about 400 m east-southeast of a major road. In the pollution rose in Figure 4C, wind direction aligning with the landing path to runway 28 at the Zurich airport coincides with high LDSA at Kloten from low to high wind speeds. The diurnal patterns at this station also reflect potential influence from both sources (Figure 4D). There was a morning increase in LDSA with the morning traffic. At night, high LDSA coincided with landing periods over the Kloten region. Highest daytime concentrations were generally on weekdays in the spring and winter. The increase in night-time concentrations in the winter and fall commenced earlier than the increase in the night-time LDSA in the summer and spring.

###### LDSA and Other PM Metrics: Kaserne and Stampfenbachstrasse

**FIGURE 5:**
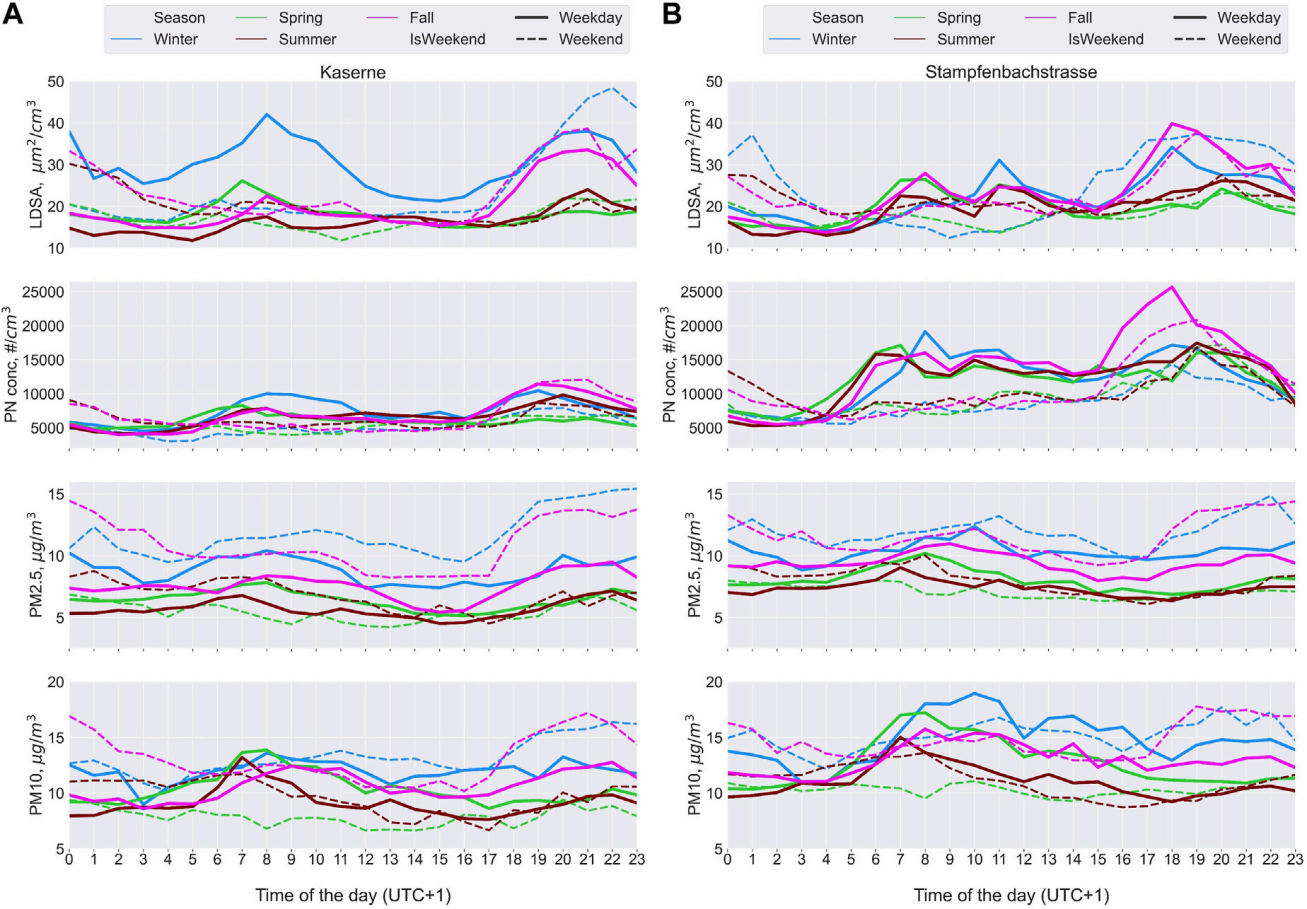
Line plots comparing the seasonal diurnal trends in particulate matter (PM) metrics (PM number concentration (PN conc); PM mass concentration of particles of diameter less than 2.5 microns (PM2.5) and 10 microns (PM10) measured at Kaserne **(A)** and Stampfenbachstrasse **(B)** on weekdays and weekends (Zurich, Switzerland. 2022).

LDSA was measured alongside PNC and PM_mass metrics at Kaserne and Stampfenbachstrasse. At Kaserne, the diurnal patterns of the different metrics were closely related, showing a small morning peak on weekdays and higher night-time concentrations on weekdays and weekends, especially in the fall and winter (Figure 5A). The diurnal patterns at Stampfenbachstrasse were different from those at Kaserne, and the metrics were not as closely related (Figure 5B). On weekdays, there were often two morning peaks in LDSA and PNC and to a lesser extent in PM10 and PM2.5. There was also a late afternoon/evening increase in LDSA and PNC, pronounced in the fall and winter. On weekends, there was a peak in the late evening in LDSA and PNC, while PM2.5 and PM10 increased through the night-time period, especially in the winter and fall.

The correlations (ρ) between LDSA and PNC (PN) at both Kaserne and Stampfenbachstrasse were high (0.8 at Kaserne and 0.68 at Stampfenbachstrasse; Supplementary Figure S10). ρ between LDSA and the mass metrics at Kaserne were also high, 0.74 with PM2.5 and 0.75 with PM10. At Stampfenbachstrasse, ρ between LDSA and PM2.5 and PM10 were lower, 0.58 with PM2.5 and 0.59 with PM10.

## Discussion

Observed LDSA in this study were comparable to previous studies in Europe and Switzerland [30, 39, 40, 42, 43, 52]. Like these studies, we observed the highest LDSA at stations close to busy roads while rural areas had the lowest LDSA. Figure 1 indicates the presence of high outliers (values greater than 1.5 times the interquartile range) which may be indicative of acute high polluting events at these locations. Though road traffic is important for LDSA, other sources and factors also contributed to the observed spatiotemporal variability of LDSA.

### Spatial Variability and Wind

Spatial variability in LDSA is driven by the sources and intensity of emissions, the distance from the sources of pollution, and meteorology [19, 53]. Like other studies, we generally observed an inverse relationship with wind speed [43] up to about 7 m/s due to atmospheric dilution (Figure 2). Local sources prevail at these low wind speeds; as wind speed increases, atmospheric dilution or long-range transport may influence concentrations [43]. In our study, it appears that long-range transport of pollution also occurred, especially when winds crossed high polluting areas. At Balsberg, high local pollution from road traffic to the west of the station was pronounced at low wind speeds. Balsberg is also about 1 km south and 0.5 km east of the Zurich airport, which may have contributed to high LDSA at high wind speeds from the west across the flight paths to and from runway 34 [35, 54].

### Temporal Variability

Like other studies, we observed a close relationship between local sources of emission and the temporal pattern of LDSA. Also of importance were meteorological factors affecting atmospheric dilution [22, 39, 43]. Lower atmospheric dilution (and low precipitation in the fall of 2021) in addition to residential combustion led to observations of higher LDSA in the colder seasons than in the summer, which was marked by high precipitation.

#### Background LDSA

Brütten, the remote station, had the lowest LDSA. The range of median background LDSA at other stations agreed with that of total LDSA at Brütten, though the means differed (*α* = 0.05, Supplementary Table S4). Background LDSA was influenced by the location; while rural night-time background LDSA remained below 20 (±2) µm^2^/cm^3^ in all seasons, other locations sometimes recorded higher LDSA. Such high background was pronounced in the winter and fall, when there may be less sufficient atmospheric dilution to restore concentrations to lower background levels.

#### Traffic Emissions and LDSA

By the roadside stations (Rosengartenstrasse and Balsberg), LDSA peaked with the morning traffic rush-hour. In the warmer seasons, LDSA decreased through the afternoon/evening rush-hour, attributed to high atmospheric dilution as has been observed in other studies [39]. In the colder seasons, LDSA remained high, with some reflection of the afternoon/evening peak in the winter. At Balsberg, winter night-time LDSA decreased after the traffic peak. At Rosengartenstrasse, LDSA was influenced by the surrounding residential area at night, staying high after the traffic peak in the winter and fall, especially on weekends [10, 30, 39, 43].

Higher median pollutant concentrations at Rosengartengstrasse (except for LDSA and wintertime PM10) than at Balsberg may be related to the traffic flow especially during peak hours [55] and residential combustion. At Rosengartenstrasse, the speed limit is 50 km/h, and traffic is more prone to being congested than on the motorway next to the Balsberg station (traffic congestion levels estimated by Google Maps [56]). The speed limit on the motorway next to the Balsberg station is regulated between 80 and 100 km/h based on the traffic volume for better traffic flow [57]. Higher frequency of acceleration and deceleration in traffic congestion has been shown to increase vehicle emissions [55]. Such pollution creates exposure hotspots for residents close to Rosengartenstrasse.

We observed seasonal differences in the correlation between LDSA and co-pollutants. High correlations between NOx and LDSA indicate traffic influence on LDSA and, like other studies [10, 22, 39, 42, 43], was highest in the winter. We observed similar correlations to other studies between NOx and LDSA (Table 1). Since the influence of wind direction was not considered, the traffic counts considered here may not reflect the true impact of traffic, hence, the low correlations with traffic. We also observed similar PM_mass correlations with LDSA to other studies (Table 1). PM10 and PM2.5 had lower correlations with LDSA due to the dominance of particles less than 100 nm in traffic PM emissions [16, 17]. The PM_mass correlations also differed with seasons between both stations; highest correlations were in the winter at Balsberg, but lowest in the winter at Rosengartenstrasse. These differences may be due to residential heating in the cooler seasons at Rosengartenstrasse [30].

**TABLE 1:** Compilation of correlations between lung-deposited surface area concentration (LDSA) and co-pollutants from this study and other studies; correlations for this study considers all stations excluding the rural stations and not separated into seasons (Zurich, Switzerland. 2022).

Study	NO	NO_2_	NOx	PM2.5	PM10
[22]		0.32–0.8		0.3–0.58	0.17–0.72
[30]		0.32–0.73		0.23–0.58	
[10]			0.35–0.75	0.3–0.36	
This Study (all stations, except rural stations)	0.44–0.65	0.59–0.70	0.55–0.68	0.46–0.64	0.44–0.66

#### Potential Airport Influence at Kloten Feld

The Kloten station is influenced by both road and air traffic, especially when wind blows from the west- northwest direction; this is visible in the pollution rose at Kloten Feld (Figures 2, 4C). The influence of road traffic is evident in the morning peak in LDSA; similar roads to those at Balsberg contribute from the west-northwest to LDSA at Kloten. The landing path of the Zurich airport’s “east approach concept,” which is between 21:00 and 23:30 on weekdays and 20:00 and 23:30 on weekends [54], is visible on the pollution rose as a trail of high LDSA in the southeast-northwest direction and as night-time peaks in LDSA (Figure 4D) when traffic is at its lowest (Balsberg, Figure 3). The earlier and broad (weekend) fall and winter peaks in night-time LDSA may be due to additional residential combustion (not present in the spring and summer); and the later peak (winter weekdays) reflects the air traffic influx. The seasonal differences in air travel are also visible (Figure 4D): summer night-time LDSA were as high or higher than winter and fall LDSA with higher summer flight movements [58]. This air traffic influence on ground air quality is similar to previous observations with PNC, showing airport influence up to 5 km from the airport and 300 m above ground [35, 59]. The question about the health impact on residents along this flight path remains open.

#### LDSA and Other PM Metrics: Kaserne and Stampfenbachstrasse

Ambient particle size distribution (PSD) changes with distance from the sources of emission, with sub-100 nm dominant close to combustion sources [16, 17, 19, 60, 61]. The presence of these particles is not reflected in PM mass metrics, but in PNC and LDSA. We observed the influence of the nature of the sampling location on different PM metrics at Kaserne and Stampfenbachstrasse, where PNC and PM_mass are also monitored. Like in other case studies, winter and fall had the highest pollutant levels.

Kaserne is a partially closed square with food trucks and is in the tourist hub of Zurich. The monitoring station here is about 50 m from the food trucks. The orientation of the buildings around the square may isolate the square from the bulk of emissions from surrounding roads and may also allow for retention and recirculation within the square [62]. Being a region for social activities, we observed some morning increase in emissions with increase in activities in the city, and especially, an increase in the evening with social and nightlife activities. Low atmospheric dilution probably drove the night-time high concentrations in the fall and winter.

At Stampfenbachstrasse, the LDSA device was located next to a pizzeria’s kitchen window. Here, the kitchen’s morning opening hours are reflected in the second morning LDSA peak; the start of the evening peak also coincided with the evening operating hours. A previous study has observed high PM emissions, including LDSA, in pizzerias [63]. Our data therefore also show that high-combustion eateries may create local outdoor hotspots with potential influence on indoor air quality in neighbouring residences.

Regarding other PM metrics, there was good rank correlation (ρ) between LDSA and PNC. The combination of some distance from the source of emission and recirculation at Kaserne may have allowed ageing of particles, including particle growth [62]. Such ageing allowed for good correlations between LDSA and PM_mass, and moderate correlation between PNC and PM_mass. PSD measurements will better inform the suggested particle ageing.

Stampfenbachstrasse, on the contrary, does not have the possibility for particle retention/ageing, leading to lower correlations between LDSA and PM_mass, and PNC and PM_mass. PM_mass measurements were about 10 m from the pizzeria. The correlation between LDSA and PNC was good, though lower than at Kaserne, as LDSA still misses particles less than 20 nm [28, 29, 41]. There were also differences in the diurnal patterns of the different metrics; both LDSA and PNC clearly captured the kitchen’s schedule unlike the mass metrics. These data show that mass metrics alone here may miss the high exposure of nearby residents.

### Conclusion

We presented LDSA from a network of ten devices in different environments in Zurich, showing significant differences temporally, and between the stations. The spatial and temporal heterogeneity was strongly influenced by the different local sources of emissions and meteorology. We observed the influence of road traffic, a restaurant, and an airport on ambient LDSA. Close to the city center and in residential areas, residential and social activities also contributed to high night-time LDSA (with the added effect of low atmospheric dilution). In addition, LDSA was better correlated with PNC than with PM_mass, reflecting the timely changes in PNC, not evident in PM_mass. As PNC measurements are expensive, LDSA measurements by diffusion chargers may serve as a good alternative for cost-effective UFP exposure monitoring.

We tested the prototypes of an LDSA sensor specifically designed for application in a network. Of the ten sensors, only one had a major problem with significant data loss after a year’s deployment. The sensors have been further optimized. Despite no maintenance over a year, the precision of the devices and qualitative agreement with a CPC was maintained. These sensors are promising for improving the data availability of LDSA exposure and could provide a basis for more statistically significant epidemiological studies with LDSA, and improved LDSA and UFP exposure modelling. LDSA reflects the reactive surface area of PM deposited in the lungs; with better epidemiological studies, there is a potential for better quantification of the impacts of ambient UFP and their regulation.

Going forward, we intend to expand this LDSA network within and beyond Switzerland, as well as performing PM characterization at multiple sampling locations. Data from this and future deployments will be applied in a more detailed assessment of LDSA trends, contributions from different sources, and will feed the development of a high-resolution spatiotemporal LDSA model. The obtained high-resolution data will then be applied in an epidemiological study to assess the relationships between PM exposure as LDSA and health outcomes.

## References

[1] United States Environmental Protection Agency (EPA). Setting and Reviewing Standards to Control Particulate Matter (PM) Pollution (2022). Available from: https://www.epa.gov/pm-pollution/setting-and-reviewing-standards-control-particulate-matter-pm-pollution#review (Accessed April 1, 2022).

[2] Swiss Federal office of the Environment (FOEN). Immission Limit Values of the Air Pollution Control Ordinance (2022). Available from: https://www.bafu.admin.ch/bafu/de/home/themen/luft/fachinformationen/luftqualitaet-in-der-schweiz/grenzwerte-fuer-die-luftbelastung/immissionsgrenzwerte-der-luftreinhalte-verordnung–lrv-.html (Accessed April 1, 2022).

[3] World Health Organization. Ambient (Outdoor) Air Pollution: WHO (2021). Available from: https://www.who.int/news-room/fact-sheets/detail/ambient-(outdoor)-air-quality-and-health (Accessed September 1, 2022).

[4] Kreyling WG Tuch T Peters A Pitz M Heinrich J Stölzel M Diverging Long-Term Trends in Ambient Urban Particle Mass and Number Concentrations Associated with Emission Changes Caused by the German Unification. Atmos Environ (2003) 37(27):3841–8. 10.1016/s1352-2310(03)00457-6

[5] Lorelei de Jesus A Thompson H Knibbs LD Kowalski M Cyrys J Niemi JV Long-term Trends in PM2.5 Mass and Particle Number Concentrations in Urban Air: The Impacts of Mitigation Measures and Extreme Events Due to Changing Climates. Environ Pollut (2020) 263(Pt A):114500. 10.1016/j.envpol.2020.114500 32268234

[6] Pitz M Kreyling WG Hölscher B Cyrys J Wichmann HE Heinrich J. Change of the Ambient Particle Size Distribution in East Germany between 1993 and 1999. Atmos Environ (2001) 35(25):4357–66. 10.1016/s1352-2310(01)00229-1

[7] Ntziachristos L Giechaskiel B Ristimäki J Keskinen J. Use of a corona Charger for the Characterisation of Automotive Exhaust Aerosol. J Aerosol Sci (2004) 35(8):943–63. 10.1016/j.jaerosci.2004.02.005

[8] Chalupa DC Morrow PE Oberdörster G Utell MJ Frampton MW. Ultrafine Particle Deposition in Subjects with Asthma. Environ Health Perspect (2004) 112(8):879–82. 10.1289/ehp.6851 15175176 PMC1242016

[9] Hennig F Quass U Hellack B Küpper M Kuhlbusch TAJ Stafoggia M Ultrafine and Fine Particle Number and Surface Area Concentrations and Daily Cause-specific Mortality in the Ruhr Area, Germany, 2009-2014. Environ Health Perspect (2018) 126(2):027008. 10.1289/EHP2054 29467106 PMC6066351

[10] Joel K Heino K Niemi Jarkko V Erkka S Harri P Anu K Long-term Sensor Measurements of Lung Deposited Surface Area of Particulate Matter Emitted from Local Vehicular and Residential wood Combustion Sources. Aerosol Sci Technol (2020) 54(2):190–202. 10.1080/02786826.2019.1668909

[11] Oberdörster G. Toxicology of Ultrafine Particles: *In Vivo* Studies. Philos Trans R Soc Lond (2000) 358(1775):2719–40. 10.1098/rsta.2000.0680

[12] Oberdörster G. Pulmonary Effects of Inhaled Ultrafine Particles. Int Arch Occup Environ Health (2001) 74(1):1–8. 10.1007/s004200000185 11196075

[13] Ohlwein S Kappeler R Kutlar Joss M Künzli N Hoffmann B. Health Effects of Ultrafine Particles: a Systematic Literature Review Update of Epidemiological Evidence. Int J Public Health (2019) 64(4):547–59. 10.1007/s00038-019-01202-7 30790006

[14] Schraufnagel DE. The Health Effects of Ultrafine Particles. Exp Mol Med (2020) 52(3):311–7. 10.1038/s12276-020-0403-3 32203102 PMC7156741

[15] Hussain S Boland S Baeza-Squiban A Hamel R Thomassen LCJ Martens JA Oxidative Stress and Proinflammatory Effects of Carbon Black and Titanium Dioxide Nanoparticles: Role of Particle Surface Area and Internalized Amount. Toxicology (2009) 260(1-3):142–9. 10.1016/j.tox.2009.04.001 19464580

[16] Vu TV Delgado-Saborit JM Harrison RM. Review: Particle Number Size Distributions from Seven Major Sources and Implications for Source Apportionment Studies. Atmos Environ (2015) 122(D23):114–32. 10.1016/j.atmosenv.2015.09.027

[17] Brines M Dall’Osto M Beddows DCS Harrison RM Gómez-Moreno F Núñez L Traffic and Nucleation Events as Main Sources of Ultrafine Particles in High-Insolation Developed World Cities. Atmos Chem Phys (2015) 15(10):5929–45. 10.5194/acp-15-5929-2015

[18] Moreno-Ríos AL Tejeda-Benítez LP Bustillo-Lecompte CF. Sources, Characteristics, Toxicity, and Control of Ultrafine Particles: An Overview. Geosci Front (2022) 13(1):101147. 10.1016/j.gsf.2021.101147

[19] Manigrasso M Avino P. Fast Evolution of Urban Ultrafine Particles: Implications for Deposition Doses in the Human Respiratory System. Atmos Environ (2012) 51:116–23. 10.1016/j.atmosenv.2012.01.039

[20] Kwon H-S Ryu MH Carlsten C. Ultrafine Particles: Unique Physicochemical Properties Relevant to Health and Disease. Exp Mol Med (2020) 52(3):318–28. 10.1038/s12276-020-0405-1 32203103 PMC7156720

[21] Baldauf RW Devlin RB Gehr P Giannelli R Hassett-Sipple B Jung H Ultrafine Particle Metrics and Research Considerations: Review of the 2015 UFP Workshop. Int J Environ Res Public Health (2016) 13(11):1054. 10.3390/ijerph13111054 27801854 PMC5129264

[22] Eeftens M Phuleria HC Meier R Aguilera I Corradi E Davey M Spatial and Temporal Variability of Ultrafine Particles, NO2, PM2.5, PM2.5 Absorbance, PM10 and PMcoarse in Swiss Study Areas. Atmos Environ (2015) 111:60–70. 10.1016/j.atmosenv.2015.03.031

[23] Geiser M Kreyling WG. Deposition and Biokinetics of Inhaled Nanoparticles. Part Fibre Toxicol (2010) 7:2. 10.1186/1743-8977-7-2 20205860 PMC2826283

[24] Aguilera I Dratva J Caviezel S Burdet L de GE Ducret-Stich RE Particulate Matter and Subclinical Atherosclerosis: Associations between Different Particle Sizes and Sources with Carotid Intima-Media Thickness in the SAPALDIA Study. Environ Health Perspect (2016) 124(11):1700–6. 10.1289/EHP161 27258721 PMC5089877

[25] Schmid O Stoeger T. Surface Area Is the Biologically Most Effective Dose Metric for Acute Nanoparticle Toxicity in the Lung. J Aerosol Sci (2016) 99:133–43. 10.1016/j.jaerosci.2015.12.006

[26] Fissan H Neumann S Trampe A Pui DYH Shin WG. Rationale and Principle of an Instrument Measuring Lung Deposited Nanoparticle Surface Area. J Nanopart Res (2006) 9(1):53–9. 10.1007/s11051-006-9156-8

[27] Bau S Witschger O Gensdarmes F Thomas D. Determining the Count Median Diameter of Nanoaerosols by Simultaneously Measuring Their Number and Lung-Deposited Surface Area Concentrations. J Nanopart Res (2013) 15(12):2104. 10.1007/s11051-013-2104-5

[28] Fierz M Meier D Steigmeier P Burtscher H. Aerosol Measurement by Induced Currents. Aerosol Sci Technol (2014) 48(4):350–7. 10.1080/02786826.2013.875981

[29] Fierz M Houle C Steigmeier P Burtscher H. Design, Calibration, and Field Performance of a Miniature Diffusion Size Classifier. Aerosol Sci Technol (2011) 45(1):1–10. 10.1080/02786826.2010.516283

[30] Kuuluvainen H Rönkkö T Järvinen A Saari S Karjalainen P Lähde T Lung Deposited Surface Area Size Distributions of Particulate Matter in Different Urban Areas. Atmos Environ (2016) 136:105–13. 10.1016/j.atmosenv.2016.04.019

[31] Keskinen J Pietarinen K Lehtimäki M. Electrical Low Pressure Impactor. J Aerosol Sci (1992) 23(4):353–60. 10.1016/0021-8502(92)90004-f

[32] Tuch T Wehner B Pitz M Cyrys J Heinrich J Kreyling W Long-term Measurements of Size-Segregated Ambient Aerosol in Two German Cities Located 100km Apart. Atmos Environ (2003) 37(33):4687–700. 10.1016/j.atmosenv.2003.07.010

[33] Hussein T Puustinen A Aalto PP Mäkelä JM Hämeri K Kulmala M. Urban Aerosol Number Size Distributions. Atmos Chem Phys (2003) 4:391–411. 10.5194/acp-4-391-2004

[34] Manigrasso M Vitali M Protano C Avino P. Temporal Evolution of Ultrafine Particles and of Alveolar Deposited Surface Area from Main Indoor Combustion and Non-combustion Sources in a Model Room. Sci Total Environ (2017) 598:1015–26. 10.1016/j.scitotenv.2017.02.048 28468124

[35] Flueti E Ruf C Maraini S. Ultrafine Particle Concentrations Zurich Approach Runway 14: Flughafen Zürich AG (2019). Available from: https://www.adra-bale-mulhouse.fr/wp-content/uploads/2021/07/PUF_Study_Zurich_Approach-RV14_20191216.pdf (Accessed September 19, 2022).

[36] Cyrys J Pitz M Heinrich J Wichmann H-E Peters A. Spatial and Temporal Variation of Particle Number Concentration in Augsburg, Germany. Sci Total Environ (2008) 401(1-3):168–75. 10.1016/j.scitotenv.2008.03.043 18511107 PMC2583026

[37] Asbach C Fissan H Stahlmecke B Kuhlbusch TAJ Pui DYH. Conceptual Limitations and Extensions of Lung-Deposited Nanoparticle Surface Area Monitor (NSAM). J Nanopart Res (2009) 11(1):101–9. 10.1007/s11051-008-9479-8

[38] van Rijt SH Bein T Meiners S. Medical Nanoparticles for Next Generation Drug Delivery to the Lungs. Eur Respir J (2014) 44(3):765–74. 10.1183/09031936.00212813 24791828

[39] Reche C Viana M Brines M Pérez N Beddows D Alastuey A Determinants of Aerosol Lung-Deposited Surface Area Variation in an Urban Environment. Sci Total Environ (2015) 517:38–47. 10.1016/j.scitotenv.2015.02.049 25710624

[40] Cheristanidis S Grivas G Chaloulakou A. Determination of Total and Lung-Deposited Particle Surface Area Concentrations, in central Athens, Greece. Environ Monit Assess (2020) 192(10):627. 10.1007/s10661-020-08569-8 32901375

[41] Naneos particle solutions. Lung-deposited Surface Area (2021). Available from: www.naneos.ch (Accessed March 1, 2021).

[42] Eeftens M Meier R Schindler C Aguilera I Phuleria H Ineichen A Development of Land Use Regression Models for Nitrogen Dioxide, Ultrafine Particles, Lung Deposited Surface Area, and Four Other Markers of Particulate Matter Pollution in the Swiss SAPALDIA Regions. Environ Health (2016) 15:53. 10.1186/s12940-016-0137-9 27089921 PMC4835865

[43] Hama SML Ma N Cordell RL Kos GPA Wiedensohler A Monks PS. Lung Deposited Surface Area in Leicester Urban Background site/UK: Sources and Contribution of New Particle Formation. Atmos Environ (2017) 151:94–107. 10.1016/j.atmosenv.2016.12.002

[44] Kurihara K Iwata A Kiriya M Yoshino A Takami A Matsuki A Lung Deposited Surface Area of Atmospheric Aerosol Particles at Three Observatories in Japan. Atmos Environ (2021) 262:118597. 10.1016/j.atmosenv.2021.118597

[45] Fierz M. Data Quality Accessment of New LDSA Devices [E-Mail] (2022).

[46] Baudirektion Tiefbauamt Canton Zürich. Traffic Census Data on Cantonal Roads, Zürich [E-Mail] (2022). Available from: https://www.zh.ch/de/mobilitaet/gesamtverkehrsplanung/verkehrsgrundlagen/verkehrsdaten.html#1198469509 (Accessed February, 2022).

[47] Federal Roads Office FEDRO. Traffic Census Data on Federal Roads, Canton Zürich [E-Mail]; 2022 (2022). Available from: https://www.astra.admin.ch/astra/de/home.html (Accessed February, 2022).

[48] OSTLUFT. die Luftqualitätsüberwachung der Ostschweizer Kantone. Data query OSTLUFT: Air quality database (2023). Available from: https://www.ostluft.ch/index.php?id=datenabfragen (Accessed February, 2022).

[49] Nationale Beobachtungsmessnetz für Luftfremdstoffe. Data Query NABEL: Air Quality Database (2022). Available from: https://www.bafu.admin.ch/bafu/de/home/themen/luft/zustand/daten/datenabfrage-nabel.html (Accessed February, 2022).

[50] Stadt Zürich Gesundheits und Umweltdepartement. Particle Number Concentrations at UGZ Monitoring Stations [E-Mail] (2022). Available from: https://www.stadt-zuerich.ch/content/gud/de/index/umwelt_energie/luftqualitaet.html (Accessed February, 2022).

[51] Federal Office of Meteorology and Climatology MeteoSwiss. Meteorological Data portal for Teaching and Research (2022). Available from: https://www.meteoswiss.admin.ch/home/services-and-publications/advice-and-service/datenportal-fuer-lehre-und-forschung.html?query=idaweb (Accessed February, 2022).

[52] Buonanno G Marini S Morawska L Fuoco FC. Individual Dose and Exposure of Italian Children to Ultrafine Particles. Sci Total Environ (2012) 438:271–7. 10.1016/j.scitotenv.2012.08.074 23000716

[53] Dall’Osto M Thorpe A Beddows DCS Harrison RM Barlow JF Dunbar T Remarkable Dynamics of Nanoparticles in the Urban Atmosphere. Atmos Chem Phys (2011) 11(13):6623–37. 10.5194/acp-11-6623-2011

[54] Zurich Airport. Flight Movements - Current and Executed Flight Operations (2022). Available from: https://www.flughafen-zuerich.ch/en/company/responsibility/noise-and-sound-insulation/runway-concepts (Accessed September 5, 2022).

[55] Choudhary A Gokhale S. Urban Real-World Driving Traffic Emissions during Interruption and Congestion. Transportation Res D: Transport Environ (2016) 43:59–70. 10.1016/j.trd.2015.12.006

[56] Google Maps. Normal Traffic Flow in Zurich. Google (2023).

[57] Bundesamt für Strassen ASTRA. Geschwindgkeitsharmonisierung und Gefahrenwarnung (2022). Available from: https://www.astra.admin.ch/astra/de/home/themen/nationalstrassen/baustellen/nordostschweiz/abgeschlossene-projekte/verkehrsmanagement-der-infrastrukturfiliale-winterthur/projekt-im-ueberblick/geschwindigkeitsharmonisierung-und-gefahrenwarnung.html (Accessed August 25, 2022).

[58] Flughafen Zürich AG. Monthly Flight Movements: Flughafen Zürich AG (2022). Available from: https://www.flughafen-zuerich.ch/-/jssmedia/airport/portal/dokumente/das-unternehmen/politics-and-responsibility/noise-and-sound-insulation/monatliche-flugbewegungen_2202.pdf?vs=1 (Accessed September 9, 2022).

[59] Sintermann J Schaufelberger U Eugster R Götsch M. Ultrafeine Partikel in Kloten 2019 & 2020: Belastungssituation und Einfluss des Flugsverkehrs. OSTLUFT – Die Luftqualitätsüberwachung der Ostschweizer Kantone und des Fürstentums Liechtenstein (2021). Available from: https://www.ostluft.ch/fileadmin/intern/LZ_Information/Publikationen/Fachberichte/BE_UltrafeinePartikel_Kloten2019-2020_GeK_20210526.pdf (Accessed December 14, 2022).

[60] Longley ID Gallagher MW Dorsey JR Flynn M Allan JD Alfarra MR A Case Study of Aerosol (4.6nm<Dp<10μm) Number and Mass Size Distribution Measurements in a Busy Street canyon in Manchester, UK. Atmos Environ (2003) 37(12):1563–71. 10.1016/s1352-2310(03)00010-4

[61] Dall’Osto M Querol X Alastuey A O’Dowd C Harrison RM Wenger J On the Spatial Distribution and Evolution of Ultrafine Particles in Barcelona. Atmos Chem Phys (2013) 13(2):741–59. 10.5194/acp-13-741-2013

[62] Buccolieri R Sandberg M Di Sabatino S. City Breathability and its Link to Pollutant Concentration Distribution within Urban-like Geometries. Atmos Environ (2010) 44(15):1894–903. 10.1016/j.atmosenv.2010.02.022

[63] Buonanno G Morawska L Stabile L Viola A. Exposure to Particle Number, Surface Area and PM Concentrations in Pizzerias. Atmos Environ (2010) 44(32):3963–9. 10.1016/j.atmosenv.2010.07.002

