# Supplementary material for: Spatiotemporal Heterogeneity of Lung-Deposited Surface Area in Zurich Switzerland: Lung-Deposited Surface Area as a New Routine Metric for Ambient Particle Monitoring
Source: Int J Public Health. 2023 Jun 29;68:1605879. doi: 10.3389/ijph.2023.1605879 (PMC10338687; doi:10.3389/ijph.2023.1605879)
Supplement: Supplementary file 1 [file DataSheet1.pdf]

Journal name: International Journal for Public Health

Article title: Spatiotemporal heterogeneity of lung-deposited surface area (LDSA) in Zurich Switzerland: LDSA as a new metric for ambient particle monitoring

Supplementary tables and figures

1. Sampling locations

SI. Table S1. Locations of the monitoring stations for lung-deposited surface area concentration (LDSA) and descriptions of their surroundings. Land use features were identified on Swisstopo maps; major roads include highways, motorways and primary roads and primary connecting roads, other roads are considered minor roads; road types were identified using the ©SwissTopo TLM3D roads and tracks layer (1). (Zurich Switzerland, 2022)

Station Name	Site theme	Station surroundings	Elevation (m asl)	Station height (m)
Reckenholz (L4000)	Rural	Station is located on a office building with farms around; 270 m south of major road; 1.6 km north of minor road; the sensor is on the roof of a building	~460	~20
Brütten (remote (L4002))	Rural	Station is minimum 4 km from any major roads with farms around; a power distribution system and office of responsible supplier in close vicinity: the sensor is on the roof of a building	~597	8.5
Rümlang (L4006)	Rural	Station is next to a star gazing observatory, with a major road ~650 m S, minor road ~150 m N, and the Zurich airport ~2.4 km NE	~500	1.5
Kloten Feld (L4003)	Suburban	Station is next to sport and farm fields, residential buildings to the west with small factories within the town; ~ 400 m from major roads running N to NW, 660 m from Zurich airport (N); E to SW are buildings and a minor road; airport arrival flights fly over this location between 20:00 and 23:30 on weekends, and between 21:00 and 23:30 on weekdays	~444	4.5
Dübendorf (L4004)	Suburban	The station is located next to a research facility with offices, small wastewater treatment plant; major roads (a primary road W and motorway, N) close by; and residential areas are also within the neighbourhood; a large construction site shares borders with the station; there is also a local small airport 1.2 km E,	~432	

Schimmelstrasse (L4007)	Urban	The station is on a the side of a busy street junction with a major connecting road leading to the highway; the station is partly shielded by adjacent buildings with restaurants, offices and residential properties and next to a small parking lot, across from the station is a local train station.	~415	~3.7
Rosengartenstrasse (L4008)	Urban/major road	The station is surrounded by residential buildings, and on the side of a busy major road	~433	~2.1
Zürich-Kaserne (L4005)	Urban (City centre)	The station is in the city centre, within a partly closed square with access paths and small streets primarily for pedestrians; food trucks and mobile restaurants are often present within the square, less than 50 m from the sensor. In the neighbourhood of the square are shopping centres, restaurants, and bars as the square is within a tourist destination.	~409	
Stampfenbachstrasse (L4009)	Urban (City centre)	The station surrounded by school buildings, businesses, residential buildings, restaurants and hotels and on the side of a minor road with tram lines. The sensor was located at about 4 m above ground and next to the kitchen window of a pizzeria.	~445	~4.0
Opfikon Balsberg (L4001)	Major traffic zone	Urban area – offices, Zurich airport (N-NE), surrounded by heavily trafficked major roads (about 10 m from the roadside)	~435	4.5

2. Quality assessment: co-location measurements

SI. Figure S1 qualitatively shows the applicability of the LDSA devices in capturing the temporal variability in particle concentration during a co-location campaign before the deployment for the 2021-2022 campaign. There is very good agreement in time and changes in intensity between the LDSA devices and the particle counter, which are not depicted in the particle mass metrics. Mean LDSA in SI Figure 1 is the mean of the 10 devices deployed at one location (Kaserne) for about a month. The variability in the measurements across these devices were very small (bands depict 3 times standard deviation), with the relative standard deviation of.

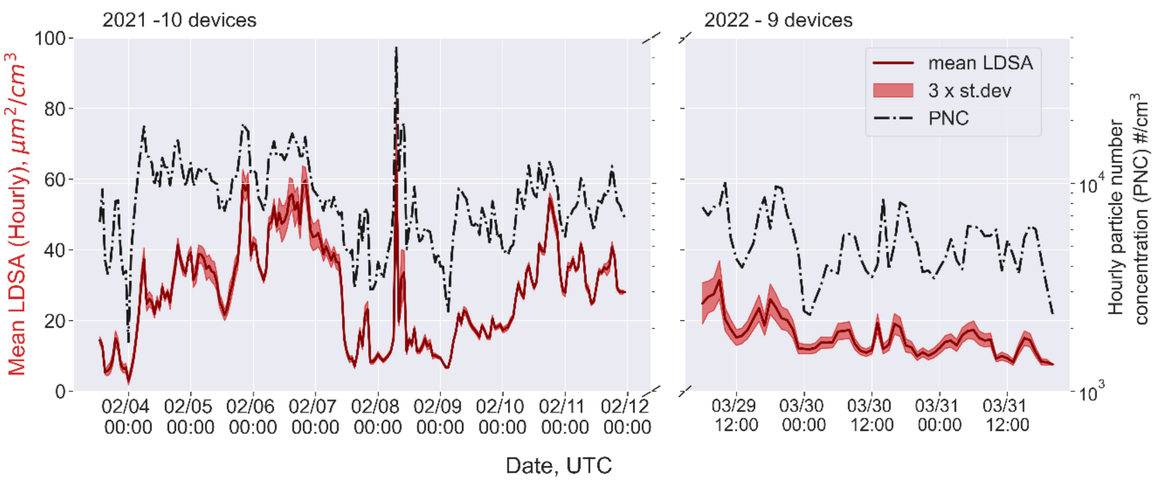

SI. Figure S1. Timeseries of the mean of hourly lung-deposited surface area for all 10 devices (mean LDSA), and particulate number concentration (PNC) during co-location measurements at Kaserne, the urban background station before the 2021-2022 deployment (left) and after (right). Three times the standard deviation across all 10 (or 9 devices after) lung-deposited surface area devices is shown as shaded bands around the mean. (Zurich Switzerland 2022)

After a year's deployment without maintenance, one device was damaged, and most devices had problems with flow readings due to clogging of flow meter. Flow within the deployed prototypes was controlled by a fan, whose fan current was monitored. With the measured fan current and calibration of the flow to the fan duty cycle, naneos developed a flow correction factor for the devices. Without this flow correction, the relative standard deviation across these devices was 7.3%; with the flow correction, the relative standard deviation was 4.3% (SI Figure 2 (right)). The prototype devices have been updated to avoid such flow problems. Previous precision was recovered upon fixing the flow issues in the devices (not shown here). SI. Table 2a and Table 2b. presents the statistics on the LDSA concentrations observed during co-location measurements before and after the long deployment.

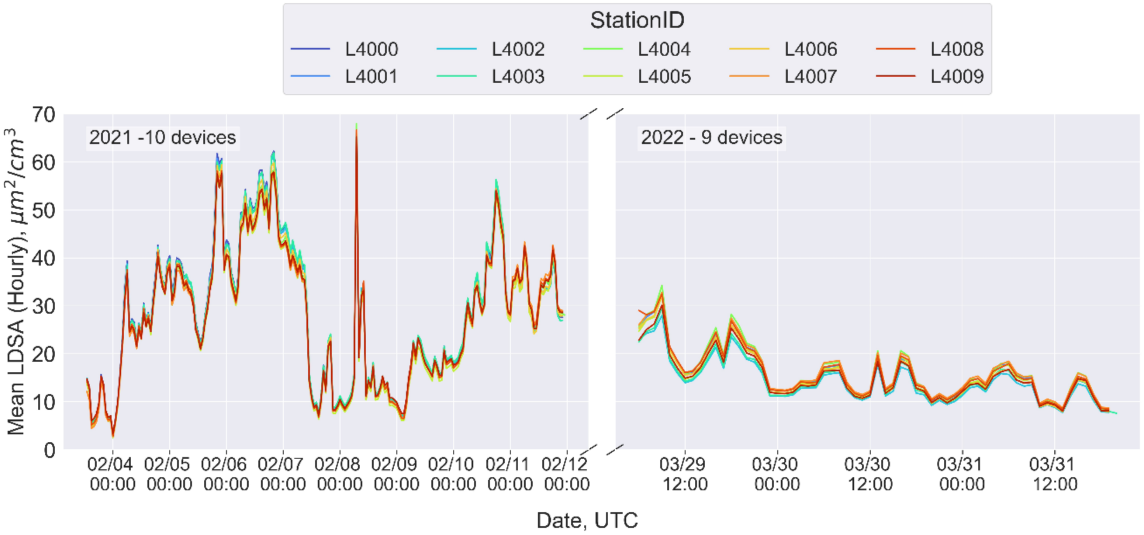

SI. Figure S2. Mean hourly lung-deposited surface area concentration (LDSA) of all ten devices co-located at Kaserne before (left) and of nine devices after the 2021 to 2022 campaign (right, flow corrections applied) (Zurich Switzerland, 2022).

SI. Table S2a. Statistics on LDSA measured during the co-location campaign before deployment of the devices to the sampling locations (Zurich Switzerland 2022)

February 2021					
**DeviceIDs**	**Mean**	**Q1**	**Median**	**Q3**	**Std. Dev**
**L4000**	29.0	16.0	28.0	39.0	17.0
**L4001**	29.0	15.0	28.0	38.0	18.0
**L4002**	29.0	16.0	28.0	38.0	18.0
**L4003**	29.0	16.0	28.0	39.0	18.0
**L4004**	28.0	15.0	27.0	37.0	18.0
**L4005**	27.0	14.0	27.0	37.0	17.0
**L4006**	28.0	15.0	27.0	38.0	18.0
**L4007**	28.0	15.0	28.0	37.0	17.0
**L4008**	28.0	15.0	28.0	38.0	18.0
**L4009**	28.0	15.0	27.0	37.0	17.0

SI. Table S2b: Statistics on LDSA measured during the co-location campaign after the 2021 to 2022 campaign (Zurich Switzerland 2022)

March 2022					
**DeviceIDs**	**Mean**	**Q1**	**Median**	**Q3**	**Std. Dev**
**L4001**	16.0	12.0	15.0	18.0	5.0
**L4002**	14.0	11.0	13.0	16.0	4.0
**L4003**	15.0	11.0	14.0	17.0	5.0
**L4004**	16.0	12.0	15.0	18.0	6.0
**L4005**	15.0	12.0	14.0	18.0	5.0
**L4006**	15.0	12.0	14.0	18.0	5.0
**L4007**	16.0	13.0	15.0	19.0	5.0
**L4008**	16.0	12.0	15.0	18.0	5.0
**L4009**	15.0	12.0	14.0	17.0	5.0

3. Data loss after clean-up, and data below the limit of detection (LOD) or above 20,000

SI. Table S3. Data counts and percent data loss after clean-up (Zurich Switzerland 2022)

Stations	Raw data count	Cleaned data count	% data Loss
**Reckenholz**	3199457	3081524	3.7
**Opfikon Balsberg**	3504760	3504679	0.002
**Brütten**	3488870	3488861	0.000
**Kloten Feld**	3503898	3500638	0.093
**Dübendorf**	3508290	3508093	0.006
**Kaserne**	3456663	3456555	0.003
**Rümlang**	3534045	3534015	0.001
**Schimmelstrasse**	3499804	3489643	0.290
**Rosengartenstrasse**	3455590	3455523	0.002
**Stampfenbachstrasse**	3522394	3522309	0.002

4. Statistical distribution of LDSA at the different stations during the 2021 to 2022 deployment

SI. Table S4. Statistical distribution of lung-deposited surface area concentration (LDSA) at the different stations during the 2021 to 2022 campaign after all data preparation steps (Zurich Switzerland, 2022)

Station	Mean	Standard _dev	Q1	Medians	Q3	Q90	Mins	Maxes
**Reckenholz**	25	14	15	21	31	44	2	127
**Opfikon Balsberg**	33	21	20	29	41	57	2	236
**Brütten**	15	7	10	14	19	24	2	120
**Kloten Feld**	26	19	14	21	32	49	2	262
**Dübendorf**	22	15	12	18	29	42	2	248
**Kaserne**	22	14	12	18	27	39	2	184
**Rümlang**	20	12	11	17	25	35	2	243
**Schimmelstrasse**	25	14	16	23	32	43	2	218
**Rosengartenstrasse**	29	14	19	27	36	46	2	147
**Stampfenbachstrasse**	22	14	13	20	28	38	2.0	317

5. Weather (temperature and precipitation), seasons and LDSA

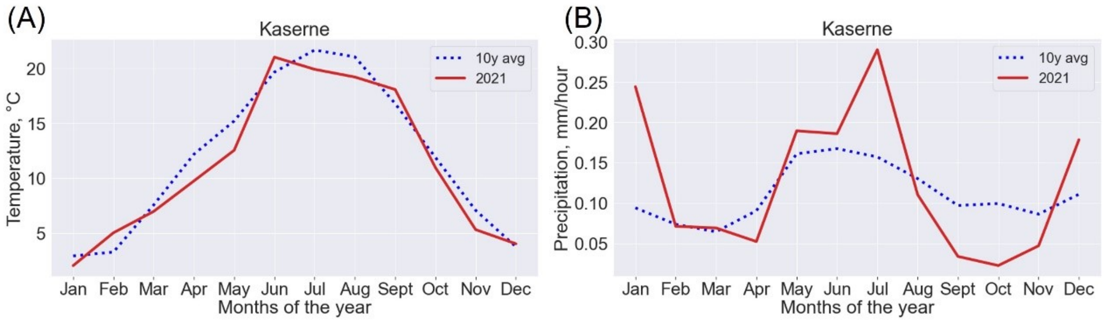

SI. Figure S3. Monthly mean temperatures (A) and monthly mean precipitation per hour (B), comparing 2021's observations to the 10-year average from 2010 to 2020 (right) at the Kaserne station. (Zurich Switzerland 2022)

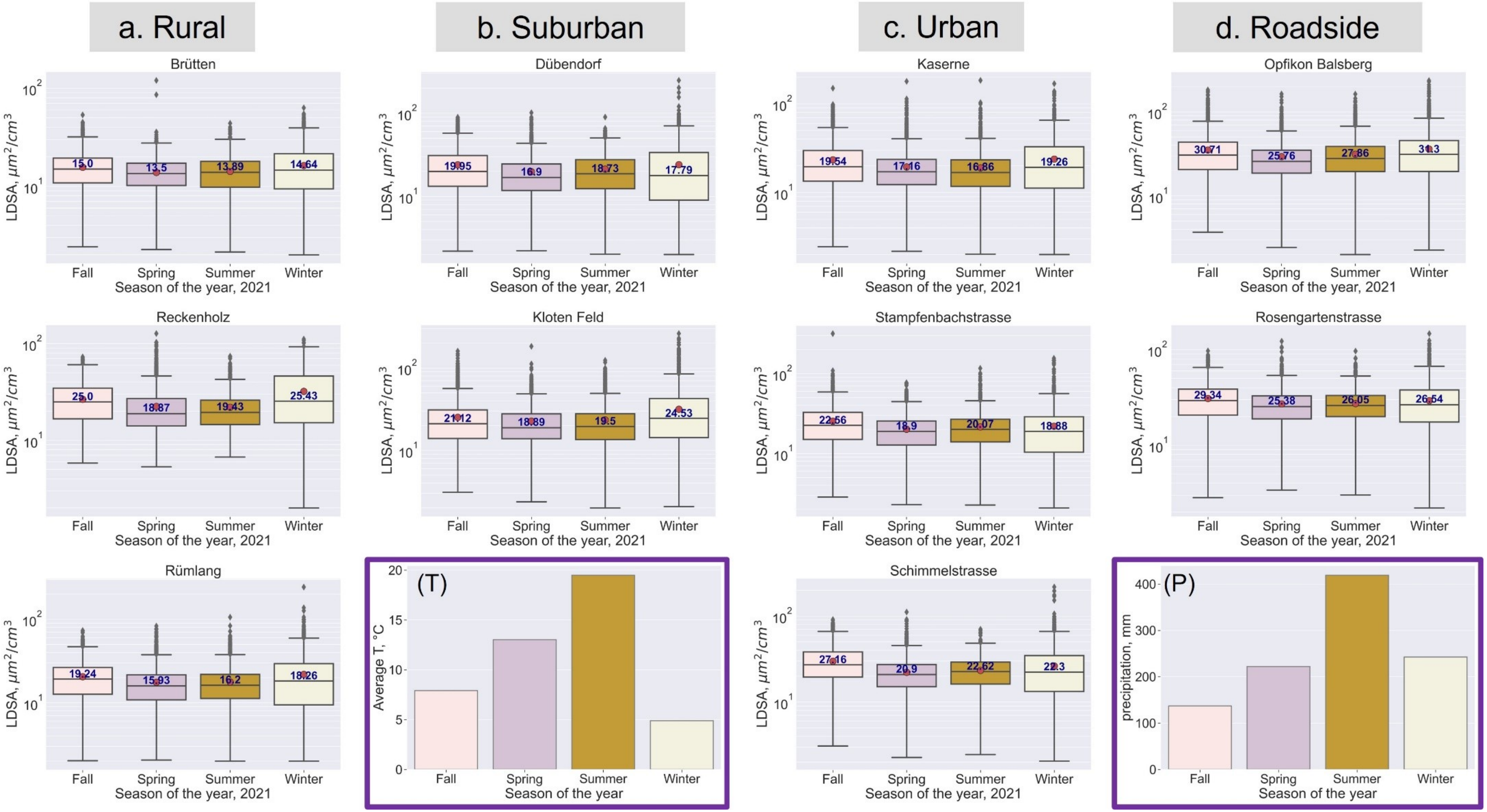

*SI. Figure S4. Statistical distribution of lung-deposited surface area concentration (LDSA) in each season at each station (**a**. Rural, **b**. Suburban; **c**. Urban, **d**. Roadside); the midlines and the numbers are the medians; the red circles are the means; the grey diamonds are the outliers, which are beyond 1.5 times the interquartile range (1.5 IQR shown as whiskers);Outlined bar plots: Bar plots showing the average temperature in each season at Kaserne (T); Bar plots showing total precipitation in each season at Kaserne (P). (Zurich Switzerland 2022)*

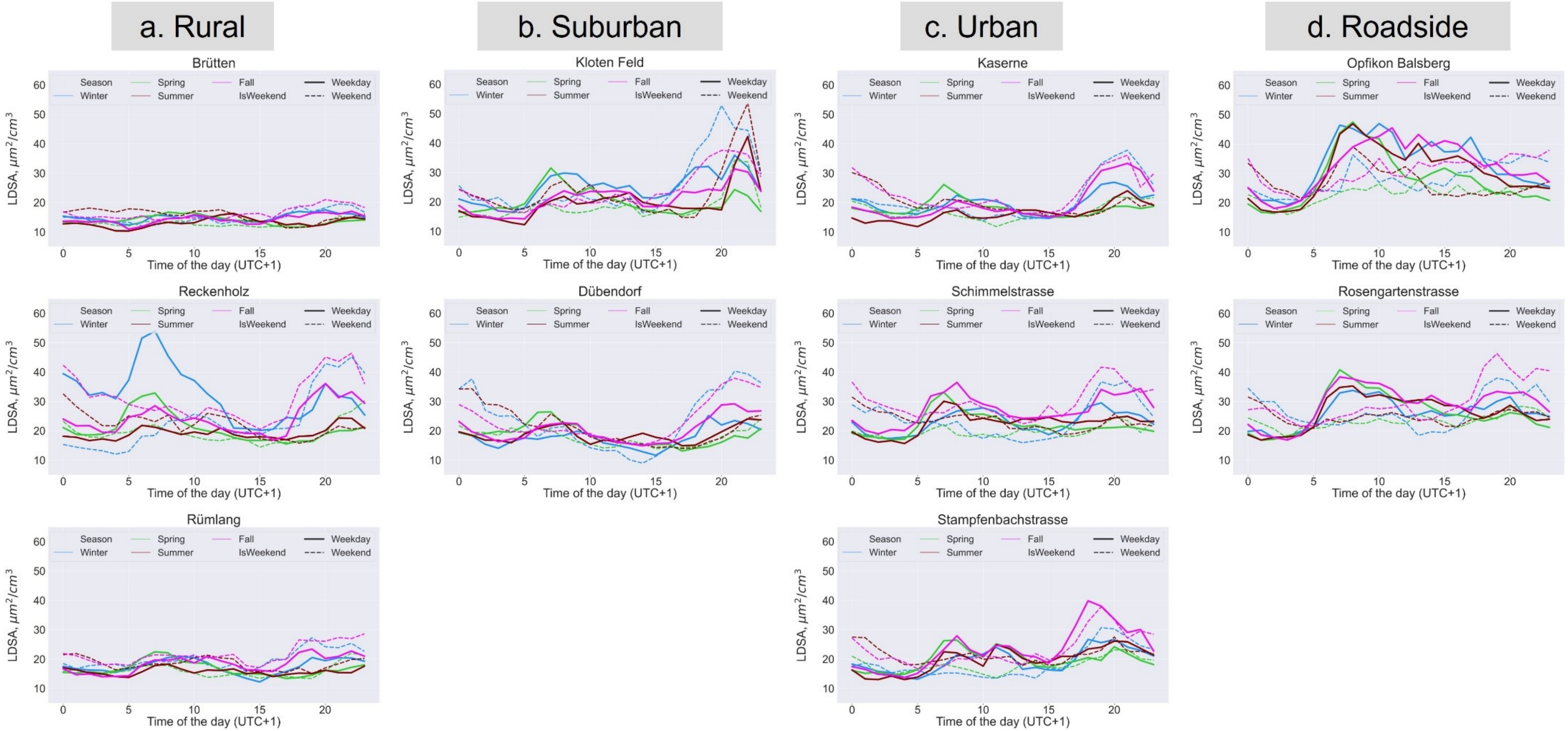

SI. Figure S5. Seasonal diurnal pattern in median lung-deposited surface area concentration (LDSA) at the different stations in the different types of environment; lines are colored by the seasons and styled by weekday or weekend: a. Rural, b. Suburban; c. Urban, d. Roadside (Zurich Switzerland 2022)

6. Background LDSA

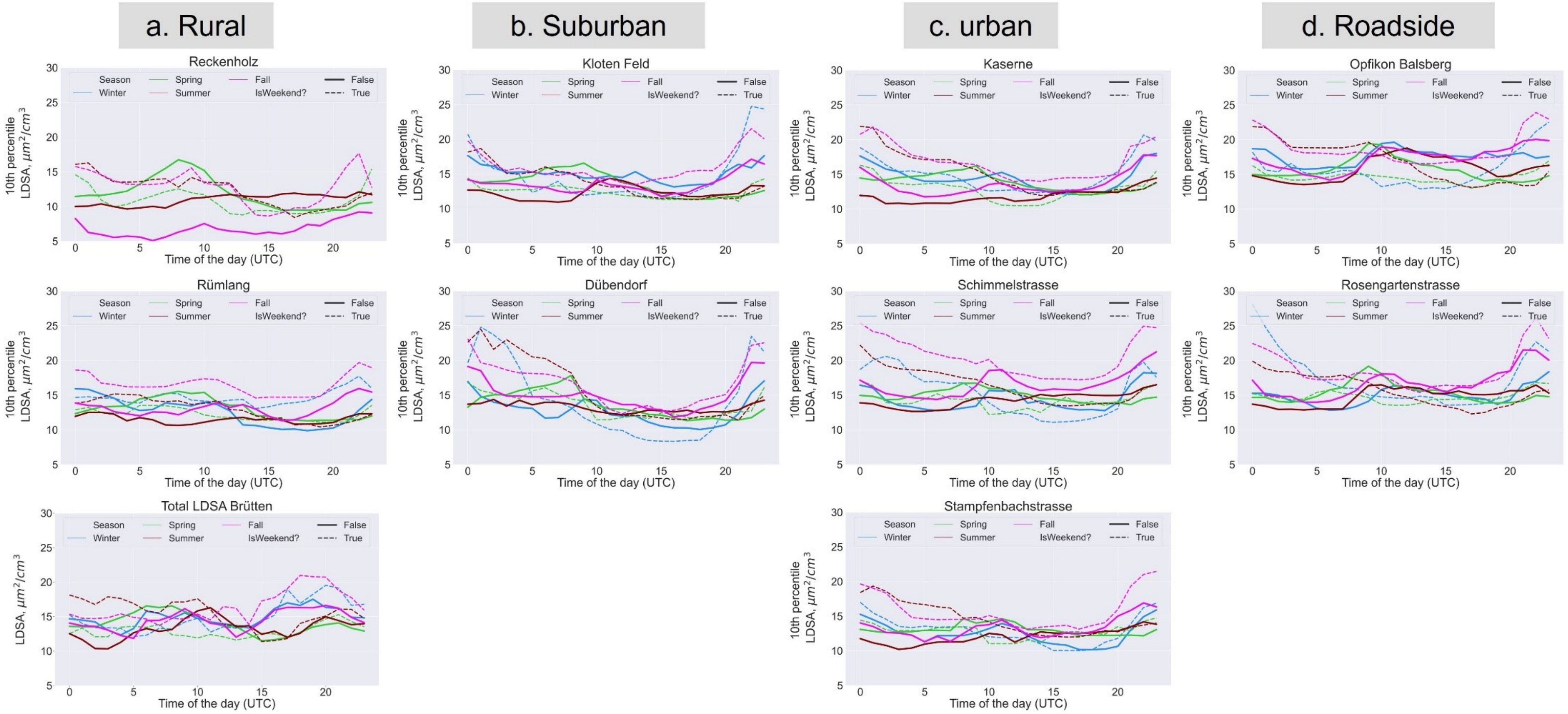

SI. Figure S6. Seasonal diurnal patterns of median background (as rolling 10th percentile over 6 hours) lung-deposited surface area concentration (LDSA) at each station, and median of total LDSA at Brütten, the regional background (bottom left). Due to damage to the instrument, data in the winter at Reckenholz (top left) were not reliable (Zurich Switzerland 2022).

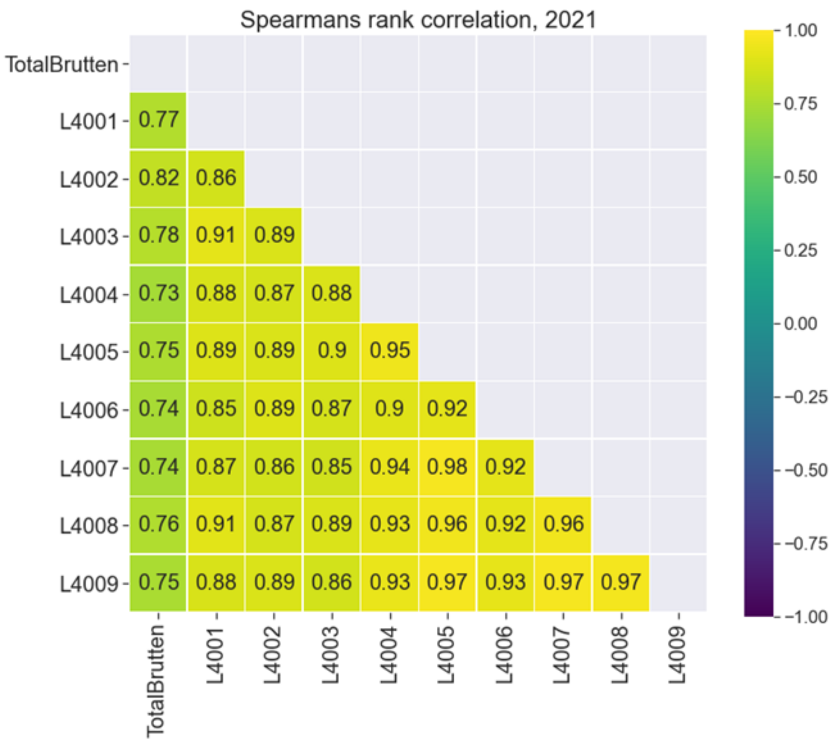

SI. Figure S7. Spearman's rank correlations between total lung-deposited surface area concentration (LDSA) at Brütten and background LDSA (rolling 10th percentile over 6 hours) at each station (See SI Table 4 for station id and names). (Zurich Switzerland 2022)

SI. Table S5. T-test statistics comparing the mean of total LDSA at Brütten, the remote station, and the background (rolling 10th percentile over 6 hours) lung-deposited surface area concentration (LDSA) at each station. (Zurich Switzerland 2022)

ID	Stations	t_statistic	p_value
L4000	Reckenholz	7.10	0.00
L4001	Opfikon Balsberg	-23.9	0.00
L4002	Brütten	35.0	0.00
L4003	Kloten Feld	-5.15	0.00
L4004	Dübendorf	-4.80	0.00
L4005	Kaserne	-2.81	0.01
L4006	Rümlang	8.06	0.00
L4007	Schimmelstrasse	-12.4	0.00
L4008	Rosengartenstrasse	-15.5	0.00
L4009	Stampfenbachstrasse	7.69	0.00

7. Traffic, LDSA and traffic co-pollutants

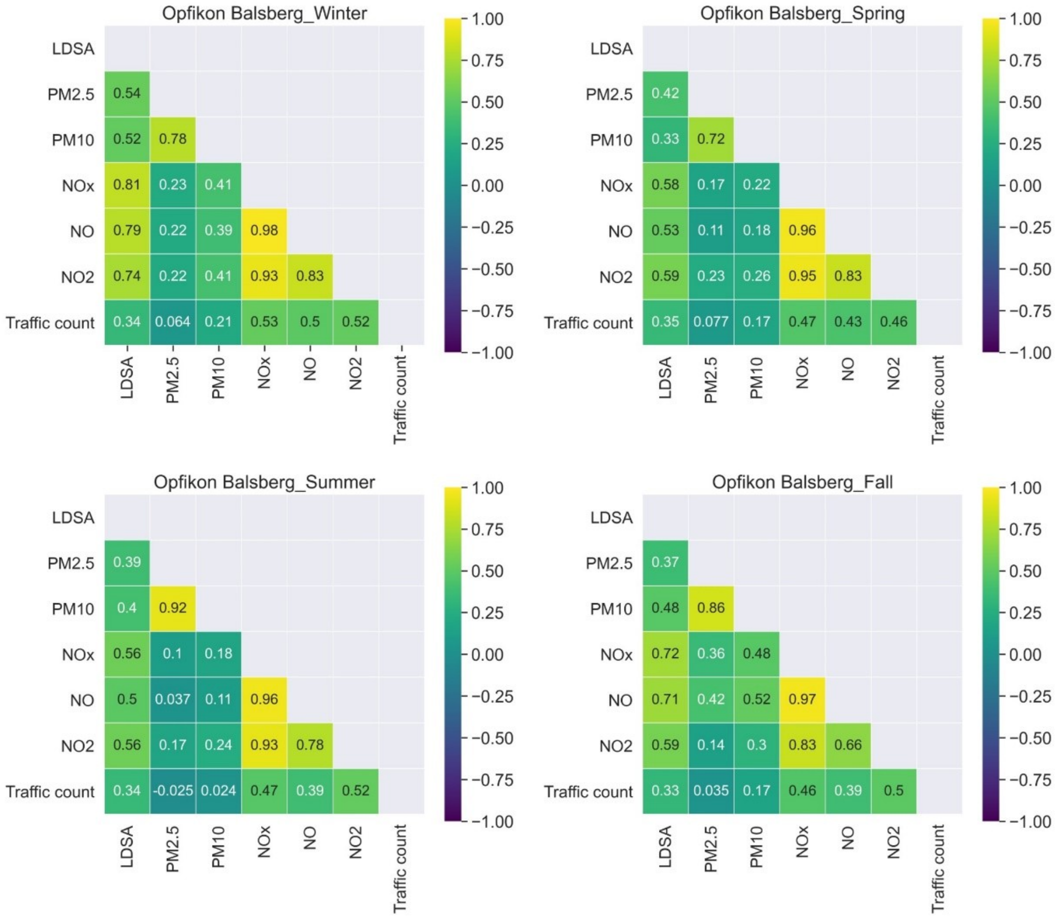

SI. Figure S8. Seasonal Pearson's correlations between traffic counts and traffic pollutants, including lung-deposited surface area concentration (LDSA), in 2021 at Opfikon Balsberg (Zurich Switzerland 2022)

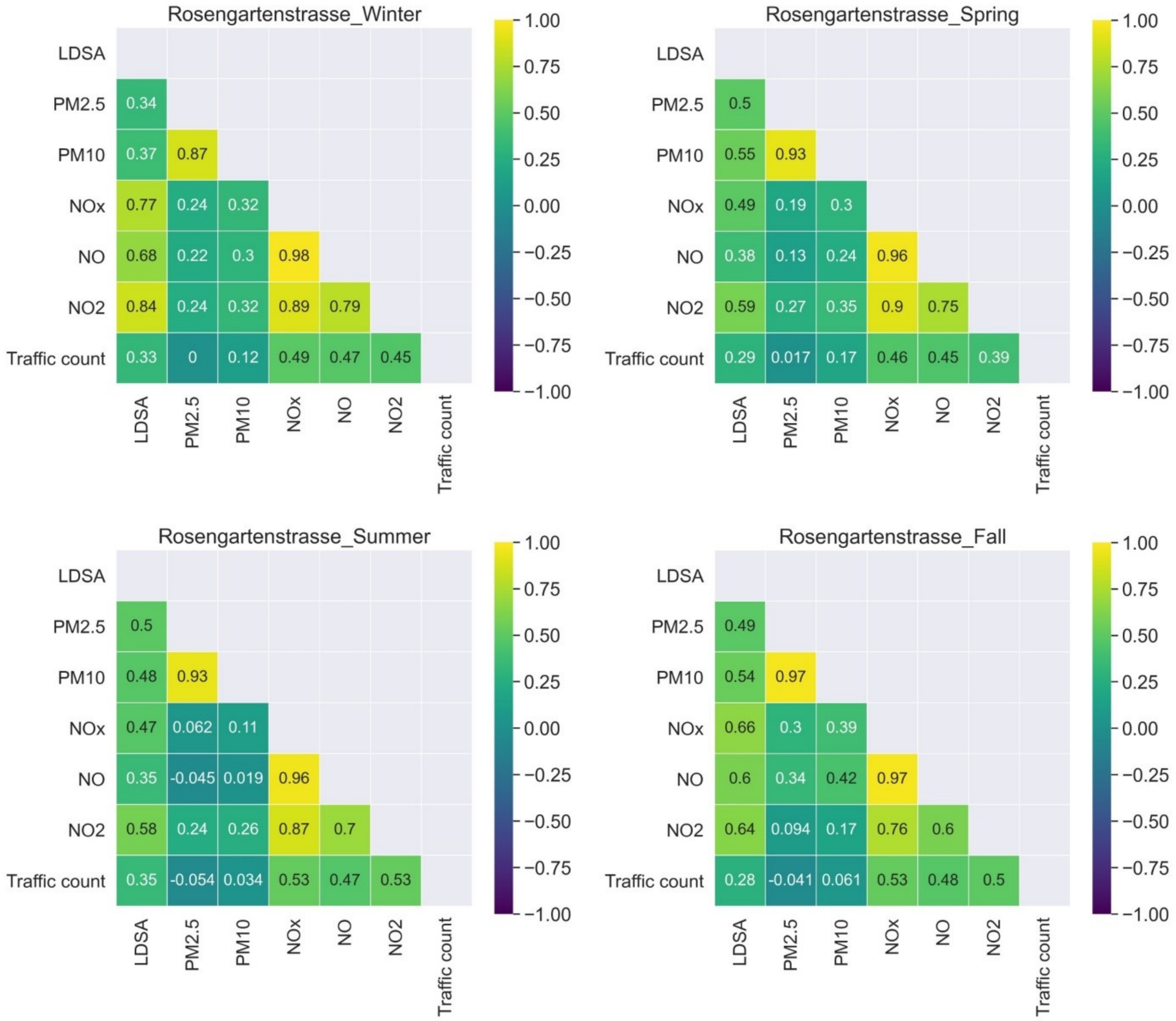

SI. Figure S9. Seasonal Pearson's correlations between traffic counts and traffic co-pollutants, including lung-deposited surface area concentration (LDSA), in 2021 at Rosengartenstrasse (Zurich Switzerland 2022)

8. LDSA and other PM metrics

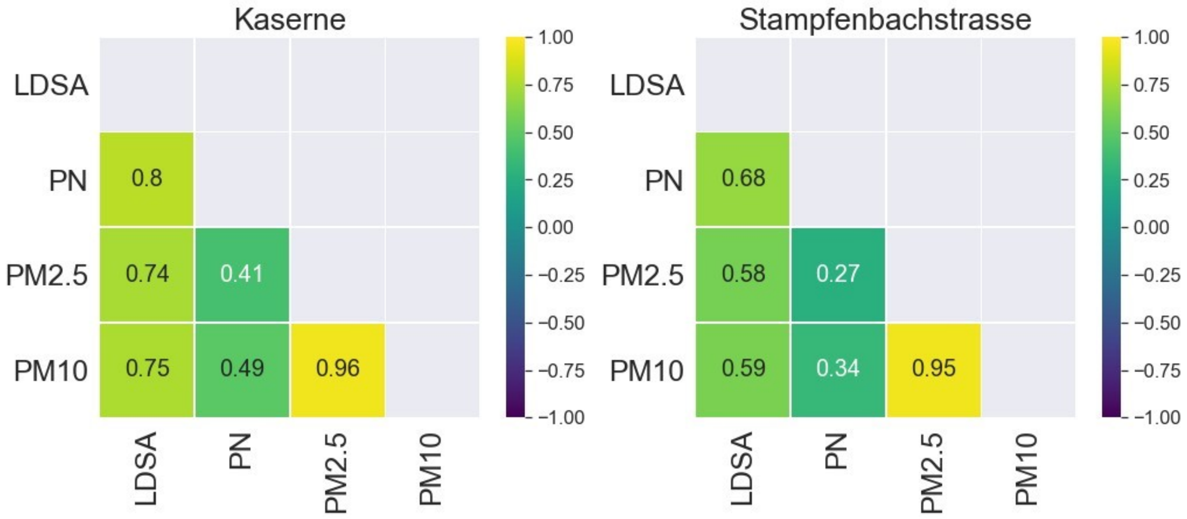

SI. Figure S10. Spearman's rank correlations between lung-deposited surface area concentration (LDSA) and other particulate matter (PM) metrics (PN = PM number concentration; PM2.5 and PM10 = PM mass concentration for particles of diameter less than 2.5 microns and 10 microns, respectively) measured at Kaserne and Stampfenbachstrasse. (Zurich Switzerland 2022)

Reference

1. Swiss TLM3D roads and tracks. Federal Office of Topography swisstopo; 2022.
